# Outcomes of resection and non-resection strategies in management of patients with advanced colorectal cancer

**DOI:** 10.1186/1477-7819-7-28

**Published:** 2009-03-10

**Authors:** Martyn D Evans, Xavier Escofet, Sharad S Karandikar, Jeffrey D Stamatakis

**Affiliations:** 1 Department of General Surgery, Princess of Wales Hospital, Bridgend, UK; 2 Department of Surgery, University Hospital of Wales, Cardiff, UK; 3 Department of Surgery, Heartlands Hospital, Birmingham, UK

## Abstract

**Background:**

The management of patients with surgically incurable bowel cancer at presentation is controversial. The aims of treatment are to optimise quality of life and prolong survival. It has been believed that the most effective palliation is achieved by resection of the primary cancer in order to pre-empt future complications. This study reviews and compares the outcomes of patients with incurable bowel cancer managed by resection and non-resection strategies over a 7-year period in a single District General Hospital.

**Patients and methods:**

All patients with surgically incurable bowel cancer at presentation were identified from the prospectively collected local ACPGBI database. Survival, using Kaplan-Meier method and log-rank test, was compared between patients managed by resection of the primary, non-resectional intervention (surgery, stent & oncological treatments) and those managed with supportive care only. The primary endpoint of the study was survival on an intention to treat basis, compared using Kaplan-Meier and log-rank tests.

**Results:**

Of 646 consecutive newly diagnosed bowel cancer patients over a 7 year period 154 cases (24%) were deemed surgically incurable at presentation. Of these surgical resection was carried out in 45 patients (29%), non-resectional intervention was followed in 52 patients (34%) and supportive treatment alone in 57 patients (37%). Median survival of each group was as follows: resected patients 11 months (I.Q range 3–18 months), non-resectional intervention 7 months (I.Q range 2–15 months) and supportive care alone 2 months (I.Q range 1–8 months). Only one patient (2%) managed by non-resectional intervention required later surgery to treat primary tumour related complications. Survival was not significantly different between resection and non-resection treatments. The overall operative mortality for the resection group was 16% (7/45 cases), with an elective mortality of 14% (4/28 cases) and emergency mortality 18% (3/17 cases).

**Conclusion:**

In an unselected bowel cancer population surgical resection of the primary tumour in patients presenting with incurable disease does not improve survival and is associated with a high risk of post-operative mortality.

## Background

Colorectal cancer (CRC) is one of the most common malignancies in the Western world. In the UK approximately 34000 patients have newly diagnosed colorectal cancer each year[1]. Between 20–30% are found to have synchronous distant metastases at the time of diagnosis [2,3]. A small, select group will be suitable for resection of hepatic metastases whereas the remaining majority are deemed surgically incurable. The aim of treatment in the majority of patients with advanced disease is palliation with a view to optimise quality of life (QoL) and survival time[4].

Patients diagnosed with Stage IV disease CRC present a common clinical dilemma. It has been recommended that optimal palliation can be achieved by resection of the primary, in order to pre-empt potential complications such as obstruction, perforation or haemorrhage, and possibly prolong survival [5,6]. However surgery, even in a non-urgent situation, carries significant risks of mortality and many patients with stage IV disease may die from progressive systemic disease before the development of any primary tumour specific complication [7,8].

The aim of this retrospective study is to review the impact of non-operative management of advanced CRC in an unselected, consecutive series of patients presenting with newly diagnosed disease. It reviews and compares the outcomes of patients with advanced bowel cancer treated with different treatment strategies, in a single colorectal unit, over a 7-year period.

## Patients and methods

Patients diagnosed with primary CRC between January 1999 and April 2006 were identified from the prospectively collected information held in a local copy of the Association of Coloproctology of Great Britain and Ireland colorectal cancer database. All patients underwent colonic imaging (Barium enema or colonoscopy), and staging with Computerised Tomography of the abdomen and chest x-ray. Patients diagnosed with rectal carcinoma, who were otherwise fit for surgery, also underwent Magnetic Resonance Imaging of the pelvis. Data collected included demographic data, ASA score of operated patients, stoma rates in operated patients and the indications for surgery in emergency patients. Patients deemed surgically incurable at presentation were studied. Patients with metastatic liver disease in whom curative treatment was carried out (primary tumour and hepatic resection) were excluded in this study.

The management plan for all patients, other than those treated by emergency surgery, was agreed at the weekly multi-disciplinary team meeting. The three treatment options for discussion with the patient were: resection of the primary lesion (resection group), non-resectional treatment which included non-resectional surgery, the use of self-expandable metallic stent and oncological treatment alone (non-resection group) and patients receiving symptomatic treatment only (supportive group). Advice regarding surgical resection versus non-resection treatment was based on two factors: presence of symptoms (bleeding, perforation or obstruction), and fitness for surgery. Those patients who were unfit for any active intervention or who presented with terminal disease were managed with supportive care. The study end point was survival on an intention to treat basis. Kaplan-Meier method and log-rank test were used to compare survival between the sub-groups and Mann-Whitney U test used to compare demographic data.

## Results

A total of 646 consecutive newly diagnosed colorectal cancer patients were identified from the database during the study period, 166 (26%) of whom were identified as stage IV at presentation. Of the patients with stage IV disease 12 (7%) had liver metastases at presentation and underwent potentially curative liver resection, so are excluded from further analysis. 154 (93%) of stage IV patients were diagnosed with advanced, surgically incurable disease at presentation, based on clinical examination and CT scan findings. Forty-five patients (29%) had a surgical resection of the primary tumour, fifty-two patients (34%) had active non-resectional treatment and fifty-seven patients (37%) received supportive care alone.

In patients with stage IV disease, 145 have died during the study period with an overall median survival time of 5 months (interquartile range 1–14 months). 2 patients treated with resection are alive at 16 and 17 months post resection, 5 patients treated with chemotherapy are alive at 11, 15, 23, 28 and 29 months post diagnosis and 2 patients managed with supportive care alone are alive at 9 and 17 months. Overall follow up is as follows: 145 patients were followed until death and the remaining 9 patients for a median of 17 months (range 11–29 months). The age and median survival by treatment modality utilised is summarised in table 1.

**Table 1:** Survival by treatment modality

	**Median Age (range)**	**Number of cases**	**Median survival (months)**	**Interquartile range (months)**	**Log rank p value against resection**
**Surgical resection of primary**	72 (26–90)	45	11	3–18	-

**Non-resection intervention**	70 (44–93)	52	7	2–15	p = 0.2056

**Supportive Care**	79 (38–95)	57	2	1–8	p = < 0.0001

**Figure 1:**
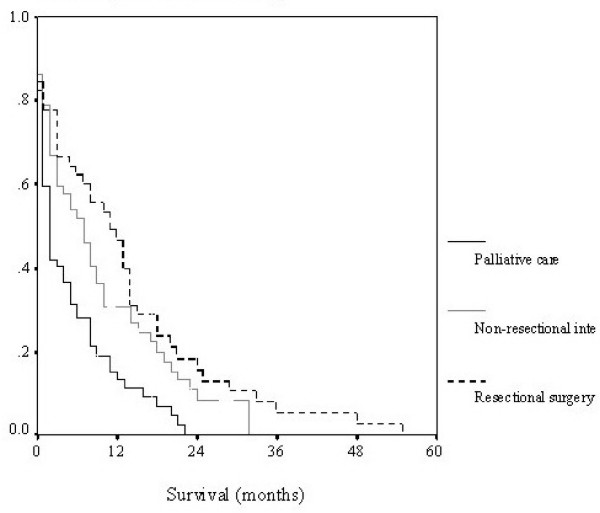
**Survival by treatment modalities**.

Patients treated by resection of the primary had the longest survival but this was not significantly longer than those treated by active, non-resectional intervention (p = 0.2056) but was significantly greater than the group treated with supportive care alone (p < 0.0001), Kaplan-Meier curve figure 1. The median age of patients undergoing resection was significantly lower than those treated with supportive care (p = 0.026) but not different from those offered active non-resectional treatment (p = 0.575). Of those patients undergoing resection, 28 were performed electively and 17 as an emergency (12 for bowel obstruction and 5 for faecal peritonitis). No difference in survival was observed between patients operated electively and those having emergency resection (median survival elective 12 months, IQ range 3–18, versus emergency resection median survival 10 months, IQ range 1–18, log-rank p = 0.95). Of those patients having elective resection 71% were ASA grade I or II. Of those patients having emergency resection 29% were ASA grade I or II. Stoma rates were 32% (9/28) in elective cases and 53% (9/17) in the emergency setting. The operative mortality in patients undergoing elective resection was 14% (4/28) patients and 18% (3/17) in patients having an emergency resection. No difference in age or survival was seen when elective and emergency resections were compared.

Non-resection intervention treatments included non-resection surgery (defunctioning stoma/bypass), chemotherapy, radiotherapy and stent. Table 2 summarises the number of cases, median age and survival for each modality. The operative mortality for non-resection surgery, stoma formation or bypass, was 36% (5/14). Of the 52 patients initially treated without resection only 1 patient underwent abdominal surgery prior to death – faecal diversion 47 months following diagnosis and treatment with chemotherapy. Two further patients had radiographic evidence of bowel obstruction, at 6 and 18 months post diagnosis, but neither underwent surgery prior to death. 9 patients required blood transfusion to treat symptomatic anaemia (1 patient required 5 admissions for transfusion, 2 patients required 2 admissions and 6 patients required a single admission).

**Table 2:** Summary of non-resectional intervention

	**Median Age (range)**	**Number of cases**	**Median survival (months)**	**Interquartile range (months)**
**Non-resectional surgery**	71 (44–87)	14	2	0–7

**Chemotherapy alone**	65 (49–75)	22	9	3–20

**Radiotherapy**	84 (66–93)	4	2	1–10

**Stent**	80 (57–92)	12	3	1–8

## Discussion

The management of patients with stage IV CRC with unresectable secondary disease remains challenging. Individual treatment needs to be tailored to optimise QoL and survival taking into account the side effects and risks of any active intervention. In patients that present requiring emergency surgery, due to perforation or bleeding, the decision is usually, although by no means always, straightforward. However in those patients presenting with non-distressing symptoms, does resection of the primary tumour offer a survival benefit and does resection prevent the onset of symptoms due to tumour complications?

This non-case-matched study has compared the survival of patients treated with resection of their primary against non-resection intervention and supportive care on an intention to treat basis. It has found that patients who undergo resection of their primary disease have the longest survival, however, this was not significantly better than those patients having active non-surgical treatment (p = 0.21). Patients treated with supportive treatment alone only were observed to have significantly worse survival than those treated by primary resection and non-resection intervention (p < 0.001).

Recommendations on management of elective cases in this series were made to patients based on multi-disciplinary team discussion that would have been guided by patient symptoms and fitness, metastatic burden of disease and patient choice. Patients undergoing surgery were either symptomatic from their disease or physiologically fitter than those patients treated with non-resectional measures, confirmed by 71% of elective surgery patients having an ASA status of I or II and a median age of 72. The elective operative mortality of 14% is high but given the small number of patients in this series is in keeping with the figure of 12% reported in the ACPGBI national audit[9]. This level of postoperative mortality is an important consideration when counselling patients for surgery with stage IV disease.

In this series patients managed with supportive care only experienced poor survival, the median being 2 months and 18% (10 of 57 cases) of patients surviving less than 1 month from diagnosis. This self selected group of patients treated with supportive care alone were those with very advanced disease or who were physiologically unfit to undergo any from of anti-cancer treatment.

During the course of this series there have been several changes in the management of stage IV CRC both in palliative treatments and an increased role for potentially curative hepatic resection. The number of patients considered suitable to undergo liver resection has increased as new surgical therapies have been introduced[10]. In addition new chemotherapeutic agents have been employed that have increased the feasibility of curative hepatic resection and significantly improved median survival for patients with surgically incurable CRC[11]. Therefore it is likely that some of the patients in this series and previous reported series would today be candidates for more aggressive liver surgery or use of newer chemotherapy regimens that may improve the survival of both non-resection and resection treatment groups in this series.

One of the concerns in managing patients with surgically incurable CRC without resection of the primary tumour is the risk of the patient presenting acutely with obstruction, bleeding or perforation either at the time of diagnosis or subsequently. In this series only 1 patient (2%) patient underwent surgery following an initial decision not to resect the primary tumour. This was for bowel obstruction not manageable by a stent. However a further 2 patients (4%) had radiographic bowel obstruction that was managed without surgery and 9 (17%) required blood transfusion to treat anaemia.

For patients presenting with malignant large bowel obstruction there is an increased trend in the use of self expandable metallic stents (SEMS). A recent systematic review examined the role of SEMS in this situation and showed successful palliation in 90% of 336 reported cases with technical failure reported in 8% of cases and perforation in 4% [12]. In this series the technique of stenting was introduced and developed in the unit during the study period and in the future may reduce the need for emergency surgical intervention and stoma formation in patients presenting with malignant large bowel obstruction.

Survival in metastatic CRC treated by resection and non-resection strategies are unlikely to be compared in a randomised control trial. The survival results of seven recent studies are summarised in table 3. Whether resection of the primary tumour affords a survival advantage is contentious in theses series. Some previous studies have shown a survival benefit from resection of the primary [13-15] although in each of these series the non-resection group appears to include patients who were managed with supportive care alone, which may have biased the results in favour of resection, which was a factor in our decision to divide management strategies employed, in this series, into three groups rather than two. In this series, if the survival of patients undergoing resection of their primary is compared with a combined group of patients managed with non-resectional intervention and supportive care a survival benefit is observed from resecting the primary (median 11 (IQ range 3–18) versus 3 months (IQ range 1–10 months), p = 0.006)

**Table 3:** Comparison of survival of patients treated with resection and non-resection

**Author**	**Resection**		**Non-resection**		**p value**
		
	**Number of patients**	**Median survival (months)**	**Number of patients**	**Median survival (months)**	
*Scoggins CR et al[17]	66	14.5	23 (22/23 received chemo)	16.6	0.59

*Ruo L et al[13]	127	16	103 86/103 received chemo)	9	< 0.001

*Tebutt NC et al[16]	280	14	82 all chemo	8.2	0.08 *#*

*Michel P et al[18]	31	21	23 (all chemo)	14	0.718

*Cook AD et al[14]	17658	Colon 11 Rectum 16	9096	Colon 2 Rectum 6 Chemo use not available	< 0.001

**Benoist S et al[19]	32	23	27 (all chemo)	22	0.753

*Konyalian et al[15]	62	12	47 (28/47 chemo)	4.6	< 0.0001

* non-case matched studies, ** case matched studies, *# *on multi-variate analysis

There are other potential confounding factors in some studies where the results favour resection. For example, patients treated by resection were found to have a significantly lower burden of disease in one study[13], and the impact of case selection was not recorded in the others[14,15]. Tebutt et al, have also showed improved survival in patients treated by resection against non-resection although this was not significant on multi-variate analysis (p = 0.08) although peritoneal disease, performance status, alkaline phosphotase and albumin were[16]. The remaining three studies have failed to show a survival benefit from either strategy [17-19].

Palliative chemotherapy is the only treatment modality which has been shown to improve survival of patients with surgically incurable disease[20]. Therefore it has been previously advocated that asymptomatic patients with surgically incurable disease should proceed direct to chemotherapy without resection of their primary tumour. The rationale behind this treatment strategy relates to the fact that patients are more likely to die of disease progression than any tumour specific complication and operative intervention will delay commencement of chemotherapy whilst post-operative recovery occurs [7,8,19,21].

Quality of life is of paramount importance to patients with advanced CRC and although multidisciplinary teams would consider this in tailoring individual treatment, the lack of prospectively collected QoL data, like in other similar studies, remains a limitation in this study.

## Conclusion

This non-case-matched study has shown a high risk of in-hospital mortality, with no significant survival benefit from resection of the primary, in stage IV CRC, when compared with other interventional, non-resection, treatment modalities. Non-resection strategies should be offered as part of the process of informed consent, for patients with stage IV colorectal cancer, as survival is comparable to that of resection and without the burden of a stoma. Further studies are required to assess the impact of advances in surgical oncology on QoL and survival in stage IV colorectal cancer.

## Competing interests

The authors declare that they have no competing interests.

## Authors' contributions

ME carried prepared the manuscript, carried out data analysis and collected part of the data. XE collected most of the data and carried out preliminary analysis. SK part conceived and participated in study design and helped draft the manuscript. JS part conceived and participated in study design and helped draft the manuscript. All authors read and approved the final manuscript.
